# Relieving Cellular Energy Stress in Aging, Neurodegenerative, and Metabolic Diseases, SIRT1 as a Therapeutic and Promising Node

**DOI:** 10.3389/fnagi.2021.738686

**Published:** 2021-09-20

**Authors:** Yang Fang, Xifeng Wang, Danying Yang, Yimei Lu, Gen Wei, Wen Yu, Xing Liu, Qingcui Zheng, Jun Ying, Fuzhou Hua

**Affiliations:** ^1^Department of Anesthesiology, The Second Affiliated Hospital of Nanchang University, Nanchang, China; ^2^Key Laboratory of Anesthesiology of Jiangxi Province, Nanchang, China; ^3^Department of Anesthesiology, The First Affiliated Hospital of Nanchang University, Nanchang, China

**Keywords:** silent information regulator-1, cellular energy stress, aging, age-related diseases, NAD+, AMP-dependent protein kinases, SIRT1-activating compounds

## Abstract

The intracellular energy state will alter under the influence of physiological or pathological stimuli. In response to this change, cells usually mobilize various molecules and their mechanisms to promote the stability of the intracellular energy status. Mitochondria are the main source of ATP. Previous studies have found that the function of mitochondria is impaired in aging, neurodegenerative diseases, and metabolic diseases, and the damaged mitochondria bring lower ATP production, which further worsens the progression of the disease. Silent information regulator-1 (SIRT1) is a multipotent molecule that participates in the regulation of important biological processes in cells, including cellular metabolism, cell senescence, and inflammation. In this review, we mainly discuss that promoting the expression and activity of SIRT1 contributes to alleviating the energy stress produced by physiological and pathological conditions. The review also discusses the mechanism of precise regulation of SIRT1 expression and activity in various dimensions. Finally, according to the characteristics of this mechanism in promoting the recovery of mitochondrial function, the relationship between current pharmacological preparations and aging, neurodegenerative diseases, metabolic diseases, and other diseases was analyzed.

## Introduction

Energy stress caused by increased energy demand and decreased energy supply can activate intracellular energy sensors and achieve energy homeostasis through further activation of other pathways. It is important to maintain a sufficient energy supply according to the availability of nutrients and the ability to produce ATP. ATP is produced mainly by mitochondrial oxidative phosphorylation (OXPHOS) and glycolysis in the cytoplasm which also generates a small portion (Cunnane et al., 2020). Under the low cellular ATP levels, it is urgent to take emergency measures to redistribute intracellular energy and restore it through enhancing the ability of mitochondrial biogenesis and OXPHOS which is a more efficient ATP-producing pathway than glycolysis.

SIRT1, a mammalian ortholog highly closed to Sir2, belongs to the Sirtuins family (SIRT1-SIRT7) whose function depends on nicotinamide adenine dinucleotide (NAD+) and is highly conserved in evolution (Hong et al., 2020). SIRT1 widely plays a key role in cell survival and apoptosis, inflammatory stress adaptation to cell growth, differentiation, metabolism, and senescence, and prevents mitochondrial dysfunction through regulating its transcriptional activity and protein expression levels by changing the acetylation state of substrates (Jiao and Gong, 2020; Chelladurai et al., 2021; Lou et al., 2021). In addition, SIRT1 not only catalyzes the deacetylation of lysine residues in histone substrates such as H1, H3, and H4, but also removes many acetyl groups from non-histone substrates, including peroxisome proliferator-activated receptor-gamma coactivator-1alpha (PGC-1α), forkhead box class O family (Foxos), peroxisome proliferator-activated receptor-γ (PPAR-γ), nuclear respiratory factors 1/2 (NRF1/2), liver X receptor (LXR), P53, P73, E2F transcription factor 1 (E2F1), nuclear factor-kappaB (NF-κB), and so on. Such deacetylation of transcription factors related to metabolism could lead to changes in intracellular metabolism (Ren et al., 2019; Gong et al., 2020; Maiese, 2021).

Mitochondria, which originate from prokaryotes in the endosymbiosis hypothesis, are semi-autonomous organelles that are composed of proteins encoded by transcription and translation *via* Mitochondrial DNA (MtDNA), but most protein structures are found in mitochondria are encoded by nuclear DNA (Popov, 2020). Mitochondrial dysfunction is a common feature in aging, neurodegenerative, and metabolic diseases. Therefore, mitochondria are identified as therapeutic intervention targets for many common diseases. And improvement of mitochondrial dysfunction, elimination of mutations in mitochondrial coding DNA, and mutations in nuclear gene encoding mitochondrial proteins are regarded as the three main strategies of mitochondrial therapy at present (Murphy and Hartley, 2018). Mitochondrial homeostasis depends on the balance between mitochondrial biogenesis and mitophagy. Mitochondrial biogenesis is the source of new mitochondria, which is largely regulated by transcriptional level. Mitophagy, a process that selectively clears dysfunctional mitochondria through the autophagy mechanism, is mainly mediated by PRKN (parkin RBR E3 ubiquitin protein ligase)-dependent and PRKN-independent pathways. As a molecule widely involved in the regulation of cellular processes, SIRT1 not only promotes mitochondrial biogenesis, but also regulates mitophagy, functions to clear damaged and aging mitochondria, and maintains mitochondrial quality and homeostasis (Sun et al., 2020).

In this overview article, the energy stress in some physiological and pathophysiological conditions will be discussed. And promoting the expression and activity of SIRT1 at different levels can relieve intracellular energy stress and become a promising treatment.

## Energy Stress in Multifarious Situations

Maintaining the dynamic homeostasis of cellular energy states involves a delicate balance between the consumption of energy and the generation of energy. In response to energy demands triggered by body activity and environmental stress, cells constantly adjust their metabolism, redistribute the use of energy and promote the mechanism of energy production (Herzig and Shaw, 2018).

Calorie restriction (CR) is defined as a sustained decline in energy intake compared to pre-intervention requirements without causing malnutrition (typically 20–50% less than average), and it has been shown to mediate a life-extending effect in the lower organisms through a key molecule Sir2 which previously mentioned to be highly homologous to SIRT1 in mammals (Yang N.-C. et al., 2019). The core of calorie restriction is to fully meet nutrient requirements and then gradually reduce the supply of energy-rich substrates. It can produce metabolic adaptation to reduce the metabolic rate by inducing a moderate energy deficit (Dorling et al., 2020).

The same metabolic adaptations that affect resting metabolic rate as calorie restriction were not observed in exercise-induced weight loss. This may be because exercise-induced acute energy deficits are insufficient to trigger metabolic adaptation (Karstoft et al., 2017). However, physical activity induces an acute energy deficit which activates AMP-dependent protein kinases (AMPK), uptake of substrates from plasma and lipolysis, promotes mitochondrial function and fat oxidation. Furthermore, these effects chronically improve the metabolic function of almost every organ in the body through increasing cardiorespiratory capacity, mitochondrial oxidative capacity, reducing lipid in plasma, tissues, and cells, and improving insulin sensitivity (Gao et al., 2020).

Aging is one of the powerful risk factors for the development of many diseases, particularly neurodegenerative and metabolic diseases. Alterations of metabolism caused by aging occur at different functional levels. And aging shows progressive impairment of mitochondrial respiratory function, not only displays in the antioxidant stress system but mitochondrial oxidative phosphorylation related enzymes may also be affected, results in the decrease of mitochondrial function, insulin resistance, and lipid metabolism diseases (Ma and Li, 2015), thereby reducing the production of ATP, to make the body energy stress state for a long time.

Mitochondrial homeostasis also plays a crucial role in maintaining the central function, once the homeostasis imbalance can lead to a reduction of brain energy production and activation of oxidative stress, leading to the central inflammatory response. Impaired mitochondrial function and central nervous system inflammatory microenvironment are the main characteristics of age-related neurodegenerative diseases, both of which are involved in aggravating cell energy stress. In addition, changes in energy metabolism appear to deteriorate in a gradual, regional, and disease-specific manner. Alzheimer’s disease (AD) is the most special type, and the decline in glucose metabolism begins early in the symptoms. After the onset of the disease, the activation of glial cells mediated by central nervous system inflammation increases the consumption of glucose, which further worsens the energy balance of neurons, and the persistence of neuronal energy stress further aggravates the progress of the disease (Joshi et al., 2019; Cunnane et al., 2020).

Metabolic diseases such as diabetes, obesity, and hypertension are characterized by decreased mitochondrial energy efficiency associated with impaired mitochondrial structure and function, including diminished OXPHOS, and reduced ATP production (Feng et al., 2019). Energy stress generated by insufficient energy production and metabolic substrates utilization disorder affects the physiological functions of various systems in the body which can further promote the pathological progress of these diseases (Zhang et al., 2019; Juszczak et al., 2020).

## Expression and Activity of SIRT1 Decreased in Aging, Neurodegenerative and Metabolic Diseases

As a multi-effector signaling molecule, SIRT1 can be found in both the nucleus and cytoplasm. However, there were differences in subcellular localization in different tissues and developmental stages of adult mice (Tanno et al., 2007). And existing data suggest that its subcellular localization can be altered by two nuclear localization signals (NLSs) and two nuclear export signals (NESs) which are located on the protein’s amino acid sequences (Yang T. et al., 2019). Under normal physiological conditions, the activity and expression of SIRT1 are simultaneously regulated by multiple mechanisms and maintained at a normal level to adapt to environmental stress. However, deteriorated SIRT1 expression and activity in different subcellular localization is implicated in aging, neurodegenerative and metabolic diseases.

In the last decade, researchers have found that the level of yeast Sir2 (a homolog of Sirt1) decreases as the increasing number of replications, which is one of the causes of yeast aging (Dang et al., 2009). Until now, accumulating scientific evidence indicates that the expression and activity of SIRT1 in mammalian cells decreased progressively with aging (Lamichane et al., 2019; Lin et al., 2020; Lan et al., 2021). This may be due to NAD+, the major cofactor of SIRT1, also shows a progressive decline with aging (Covarrubias et al., 2021b). Recent studies have reported a possible mechanism for this phenomenon, namely autophagy-mediated down-regulation of mammalian SIRT1 protein during aging and *in vivo* aging. As a nuclear substrate for autophagy, SIRT1 in the nucleus is recognized by autophagy protein LC3 and transferred to the cytoplasmic autophagosome for degradation (Xu et al., 2020). And study reveals that SIRT1-mediated beneficial effects could be promoted by selectively inhibiting the autophagy degradation pathway of SIRT1 in the nucleus (Wang et al., 2021).

Aging is strongly associated with the incidence of neurodegenerative diseases, particularly AD and Parkinson’s disease (PD). The decrease in serum SIRT1 protein level was more significant in patients with neurodegenerative diseases than in normal aging individuals, these results indicate the decreased expression and activity of SIRT1 are involved in the pathological process of neurodegenerative diseases (Cao et al., 2018). This is because some enzymes such as poly-ADP-ribose polymerases 1 (PARP1) are overexpressed and activated to compete with SIRT1 to utilize NAD+ during senescence, which reduces the content of NAD+ in tissues and cells, inhibits the activity of SIRT1 and its downstream pathways. And the inhibition of NAD+-SIRT1-PPARGC1A-UCP2 axis activity, resulting in increased mitochondrial membrane potential, PTEN induced putative kinase 1 (PINK1) cleavage, mitochondrial autophagy deficiency, and accelerating aging phenotype and neurodegenerative diseases, PINK1, as an autophagy-related protein attached to the outer mitochondrial membrane, is transported to the mitochondria in a membrane potential-dependent manner and degraded by mitochondrial protease, thus unable to trigger mitochondrial autophagy (Scheibye-Knudsen et al., 2014). A study has also been found that overactivation of PARP1 can also trigger the defect of mitochondrial autophagy by inhibiting the NAD+-SIRT1-PGC-1α pathway, which is related to the regulation of mitochondrial autophagy by SIRT1 through a variety of pathways, and this can be partially normalized with PARP1 inhibitors or pharmacological interventions with compounds that increase NAD+ abundance (Fang et al., 2014). There is also evidence that toxic proteins associated with neurodegenerative diseases can also reduce the expression of SIRT1, or directly interact with SIRT1 protein to inhibit its affinity with substrates and increase the acetylation of their substrates (Jiang et al., 2011; Manjula et al., 2020). Such as the expression of SIRT1 protein in the parietal cortex of patients with AD is significantly decreased and is closely related to the accumulation of Aβ and tau proteins (Kerr et al., 2017).

Metabolic diseases are a global public problem associated with overnutrition and are defined as a group of metabolic disorders including obesity, insulin resistance, type 2 diabetes, hypertension, and hyperlipidemia (Saklayen, 2018). SIRT1 is widely involved in the metabolic control of intracellular substrates in the body. The decrease of SIRT1 expression and activity is a major characteristic of metabolic diseases, and its mechanism partly explains the influence of a high-fat diet on metabolic syndrome, obese diabetes, cardiovascular disease, and other related metabolic diseases (Kosgei et al., 2020). Decrease of SIRT1 expression can also lead to lipid accumulation and impaired insulin signaling in hepatocytes. Conversely, mice on a high-fat diet to some extent avoid these diseases through the restoration of SIRT1 expression and activity (Nguyen et al., 2019; Li Z. et al., 2020).

Taken together, these data highlight the decreased expression and activity of SIRT1 as a key component of the molecular mechanisms that participate in the process of aging, neurodegenerative and metabolic diseases. However, some scientific evidence reveals that there is no inevitable connection between SIRT1 protein content and activity (Gurd et al., 2010; Ryall et al., 2015). This may be because SIRT1 is regulated by different mechanisms from multiple levels at different developmental stages, and the subcellular distribution and localization of SIRT1 also change during this process. Therefore, the accurate elaboration of the regulatory mechanism of SIRT1 in different stages of cell activity is expected to be used in the design and development of pharmacological agents for the treatment of a variety of diseases.

## The Expression and Activity of SIRT1 Are Regulated by Many Dimensions

Due to the importance of extensive participation in the regulation of energy metabolism and other cell life activities, SIRT1 activity and expression are tightly regulated in multiple dimensions. Therefore, a clear understanding of the regulatory mechanism by which SIRT1 plays a role is the basis for the development of therapeutic target drugs. In this review, we mainly attempt to expand from NAD+ to further discuss the regulation of SIRT1 expression and activity in energy metabolism.

### NAD+

SIRT1 is a key regulatory protein in life activities. Under normal physiological conditions, the activity and expression of SIRT1 are simultaneously regulated by multiple mechanisms to adapt to environmental stress. Since NAD+ is a necessary cofactor for the deacetylation activity of SIRT1, it has become a research hotspot of scientists to regulate the level of NAD+ in the cell to promote the activity of SIRT1.

As a driving force and signal molecule for energy production, NAD+ has shown that its distribution is highly differentiated. Each subcellular NAD+ pool is regulated to varying degrees and participates in various NAD+-dependent signaling mechanisms (Zhu et al., 2019). Although the overall NAD+ level will decline in the process of aging, the activity of SIRT1 is mainly dependent on the NAD+ level in the nucleus, rather than the NAD+ pool in the mitochondria or cytoplasm (Gomes et al., 2013). Sufficient experiments have shown that NAD+ levels in mitochondria and nuclei in wild-type mouse cells or tissues can be restored by supplementing NAD+ precursors such as nicotinamide riboside (NR) and nicotinamide mononucleotide (NMN). And sufficient intracellular NAD+ levels can enhance oxidative metabolism and protect mice from metabolic abnormalities induced by a high-fat diet and improve age-related pathophysiology and diseases, thereby reflecting the effect of prevention and treatment (Schöndorf et al., 2018; Yoshino et al., 2018). Promoting the activity of NAM phosphoribosyltransferase (NAMPT), the first step of the rate-limiting enzyme in NAD+ remediation synthesis, can also increase NAD+ levels. Experimental studies have found that exercise can induce an increase in NAMPT activity and promote the salvage synthesis of NAD+ (Lamb et al., 2020), and overexpression of NAMPT can also activate the SIRT1-dependent P53-CD38 pathway and SIRT1-independent NRF2-PPARα/AMPKα pathway to maintain mitochondrial content and integrity (Yu et al., 2020). However, we should consider other potential effects of enhanced NAD+ metabolism, including pro-inflammatory and cancer-promoting effects. Studies have shown that NAMPT and NAD+ play a key role in cancer. NAD+ biosynthesis is usually up-regulated in tumor cells, and increased levels of NAMPT promote pro-inflammatory aging-related secretion phenotypes (Nacarelli et al., 2019). This presents a new challenge for us to regulate the activity of SIRT1 by influencing NAD+ metabolism.

It is widely known that lactic dehydrogenase (LDH) catalyzes the redox reaction between pyruvic acid and lactic acid, and promotes the conversion of lactic acid to pyruvate under alkaline conditions. In contrast, the neutral condition promotes the reverse conversion of pyruvate to lactic acid, which can consume NADH and produce NAD+. Therefore, studies have shown that supplementing L-serine can increase the expression of mitochondrial biogenesis-related genes in C2C12 myotubes, improves the quality and function of mitochondria, and reverses insulin resistance. This is because L-serine is converted to pyruvate, which is subsequently converted to lactic acid by LDH and increases intracellular NAD+ levels, thereby activating NAD+ dependent SIRT1 activity (Sim et al., 2019).

The activation of SIRT1 by amino acids was first identified as involving leucine. Researchers found that it can increase the expression and activity of SIRT1, improve the quality and function of mitochondria, and prevent mitochondrial dysfunction and metabolic disorders in obese mice induced by a high-fat diet (Sun and Zemel, 2009). Further studies have found that leucine may not only induce the increase of NAMPT expression (Li et al., 2012), but also affect the kinetics of SIRT1, reduce the activation energy of NAD+, and have a synergistic effect with AMPK or SIRT1 activators (Zemel, 2020). Taurine, a non-protein amino acid, also can alleviate oxidative stress and apoptosis of cardiomyocytes through increasing NAD+/NADH ratio, activate SIRT1 and suppress p53 acetylation (Liu et al., 2020). SIRT1 stimulated and activated by Taurine also can alleviate the mitochondrial dysfunction induced by amyloid β 1–42 in SK-N-SH cells (Sun et al., 2014) which provides a suggestion for the diet list of patients with neurodegenerative diseases. The kynurenine pathway, the main pathway for tryptophan metabolism to produce NAD+, also regulates the activity of SIRT1 (Yin et al., 2021). Since then, the addition of amino acids to food as a method for actively regulating the function of SIRT1 has become a research hotspot.

The current view is that except nuclear sirtuins is consuming NAD+ pool, PARPs (poly-ADP-ribose polymerases) and CD38 (a NAD glycohydrolase) are competing for the same NAD+ pool, and studies have found that inhibition or knockout of PARP or CD38 can increase intracellular NAD+ levels and show an effect similar to sirtuins activation (Wang et al., 2019; Chini et al., 2020; Covarrubias et al., 2021b). The competition between PARP and SIRT1 for the NAD+ pool is age-dependent. Studies have shown that the level of NAD+ itself decreases with age and age-related diseases (Covarrubias et al., 2021a; Hikosaka et al., 2021), while the activation of PARP with age aggravates a small number of NAD+ pools. This may be because PARP appears to be related to DNA damage repair function, while aging can lead to an increase in chronic nuclear DNA damage (Shao et al., 2020).

### AMPK

Metabolic stress is characterized by a decrease in the ratio of ATP to AMP/ADP, which activates AMPK, resulting in phosphorylation that activates a series of pathways that up-regulate ATP (Herzig and Shaw, 2018; Yan et al., 2018). As a sensor for the energy state of cells, researchers found that AMPK can be activated when skeletal muscle energy is exhausted, and directly phosphorylate PGC-1α at threonine-177 and serine-538 (Spinelli et al., 2021). Moreover, researchers discovered that AMPK regulates NAD+/NADH ratio and SIRT1 activity in a manner independent of NAMPT shortly after the activation, and this process relies on mitochondrial β-oxidation (Cantó et al., 2009). Subsequently, NAMPT can be activated and involved in the regulation of NAD+ metabolism. The increased NAD+/NADH ratio directly enhanced the activity of SIRT1. However, the interaction between AMPK and SIRT1 is not a simple one-way interaction, but a more complex functional interaction. This may be due to SIRT1-mediated deacetylation of LKB1, a mammalian AMPKK, which promotes the phosphorylation and activation of AMPK subunits (Sharma A. et al., 2021).

**Figure 1:**
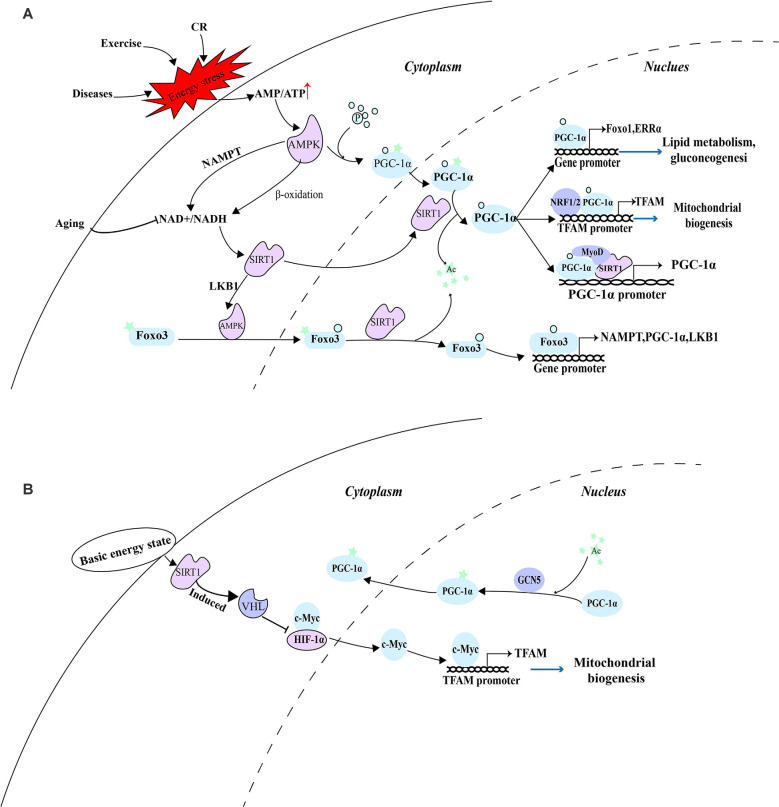
AMPK as a switch of energy state-mediated pathway. **(A)** Under a low-energy state, activated AMPK depends on the SIRT1-PGC-1α axis to eliminate cellular energy stress. **(B)** Under the basic energy state, SIRT1 regulates mitochondrial biogenesis through a pathway independent of AMPK and PGC-1α.

Consistent with this, under a low-energy state caused by exercise in skeletal muscle, AMPK was activated by the decrease in the ratio of ATP/AMP. Activated AMPK not only increases the ratio of NAD+/NADH, but also phosphorylates PGC-1α which can subsequently reduce insulin resistance, enhance glucose metabolism, and promote the recovery of energy state in skeletal muscle through improving mitochondrial biogenesis (Yang B. et al., 2021). Since the incomplete oxidation of fatty acids in skeletal muscle is caused by lipid intermediates related to insulin resistance (Koves et al., 2005), the ability of cells to maintain the oxidation of fatty acids and couple them with mitochondrial respiration is essential in the case of nutritional deficiency. A detailed explanation of their coordination mechanism can provide effective therapeutic targets for related metabolic diseases. PGC-1α phosphorylated by AMPK before deacetylation by SIRT1 may be used to distinguish which substrates are deacetylated, thus preventing the random deacetylation of SIRT1. In this case, AMPK is involved in the regulation of SIRT1 and PGC-1α and maintains its stable and sustained effect. These effectors include the activators of pyruvate dehydrogenase kinase 4 (PDK4), forkhead box O1 (FoxO1), ERRα, and PPARs gene expression. And enhanced PDK4 can phosphorylate PDH to inactivate it and prevent pyruvate from entering TCA, converting cells from glucose metabolism to lipid metabolism and then meet the energy needs of exercise (Knuiman et al., 2018). In addition, as a downstream factor activated by the SIRT1-PGC-1α axis, TFAM can play a role in diseases such as diabetes and diabetic peripheral neuropathy by enhancing the function of mitochondria, promote the complete oxidation of fatty acids in mitochondria, and provide a large number of sources of ATP for cells (Chandrasekaran et al., 2019). At the same time, the role of forkhead box O3 (FoxO3) in the SIRT1-PGC-1α axis is also very important. Activated AMPK further activates FoxO3 through phosphorylation, promoting its nuclear translocation. FoxO3 in the nucleus can be deacetylated by SIRT1, and participate in enhancing the transcription of NAMPT, PGC-1α, and LKB1, thus producing a stable and continuous role in the regulation of mitochondrial function (Sorrenti et al., 2020; Figure 1A).

The acetylation state of PGC-1α is regulated by SIRT1, histone acetyltransferase GCN5 (Mutlu and Puigserver, 2021), and steroid receptor coactivator 3 (SRC3; Hu et al., 2018). According to the availability of nutrition and the energy state of the cell, SIRT1 deacetylation and GCN5/SRC-3 acetylation jointly controls the acetylation state of PGC-1α. Under the basic energy state, AMPK is not activated and does not promote the catalytic effect of SIRT1 on PGC-1α, while GCN5 acetylates several lysine residues on PGC-1α, thus changing its location in the nucleus and inhibiting transcriptional activity (Dominy et al., 2010). The researchers discovered that hypoxia-inducible factor-1α (HIF-1α) is involved in regulating mitochondrial biogenesis and nuclear-mitochondrial communication during aging in a pathway independent of PGC-1α. In subsequent experiments, SIRT1 needs to regulate Von Hippel-Lindau (VHL) E3 ubiquitin ligase activity through post-transcriptional modification to ensure effective hydroxylation and degradation of HIF-1 in the nucleus, thus reducing the binding of HIF-1α to c-Myc, promoting the interaction between c-Myc and the binding site on the mitochondrial transcription factor A (TFAM) promoter, and enhancing the transcription and expression of mitochondrial biosynthetic genes (Gomes et al., 2013; Figure 1B).

### Regulation by Transcriptional Level

There are many binding sites of transcriptional regulatory factors in the SIRT1 promoter, therefore, SIRT1 can be regulated by multiple regulatory mechanisms at the transcriptional activity level. The transcriptional regulatory factors in this regulatory mechanism are usually the substrate of SIRT1. Researchers reported that there are two p53 binding sites on the SIRT1 promoter under basic conditions, and the P53 (activated by CBP/p300 mediated acetylation) can bind and inhibit SIRT1 gene expression, while under a low energy state, the activated FOXO3a can undergo nuclear translocation, bind and remove p53 binding to the SIRT1 promoter, then induce SIRT1 transcription and alleviate energy stress (Luo et al., 2004). At the same time, the increased expression of SIRT1 can in turn mediate the deacetylation of P53 at lysine 382, reduce its stability and activity, prevent the inhibition of SIRT1 transcription, and produce a stable and sustained effect (Vaziri et al., 2001). Consistent with this, hypoxia-induced HIF-1α and HIF-2α can directly bind to the HIF response element (HRE) on the SIRT1 promoter, increasing the transcriptional activity and expression of SIRT1 (Li T. et al., 2020). However, as mentioned earlier, the increase in SIRT1 activity and expression can destabilize and degrade HIF-1α in a VHL E3 ubiquitin ligase-dependent manner (Gomes et al., 2013). Furthermore, scientists found that the interaction between SIRT1 and FoxO1 and deacetylation can promote the auto-transcription of SIRT1 driven by FoxO1. This may be because FOXO1 could directly bind to insulin receptor substrate 1 (IRS-1) and forkhead-like consensus binding site (FKHD-L) on the SIRT1 promoter, increasing the transcription and expression of SIRT1 (Xiong et al., 2011). In response to low nutrient availability, cAMP response element-binding protein (CREB) can be activated and bind to the cAMP response element (CRE) transcriptional site on the SIRT1 promoter, thereby promoting the transcriptional activity of SIRT1 (Noriega et al., 2011). In turn, a recent study shows that CREB is also the substrate of SIRT1, and SIRT1 can deacetylate and inactivate CREB (Lu et al., 2020). In addition, as nuclear receptors, peroxisome proliferator-activated receptor (PPAR) also can increase the expression of SIRT1. Fasting for 24 h can increase the expression level of SIRT1 in mice, which may be due to the binding of PPARα to the PPAR-responsive element (PPRE) on the SIRT1 promoter (Hayashida et al., 2010). PPARβ/δ is another transcription factor, which can increase the expression of SIRT1 by binding to the Sp1 binding site in a state of starvation (Okazaki et al., 2010).

**Figure 2:**
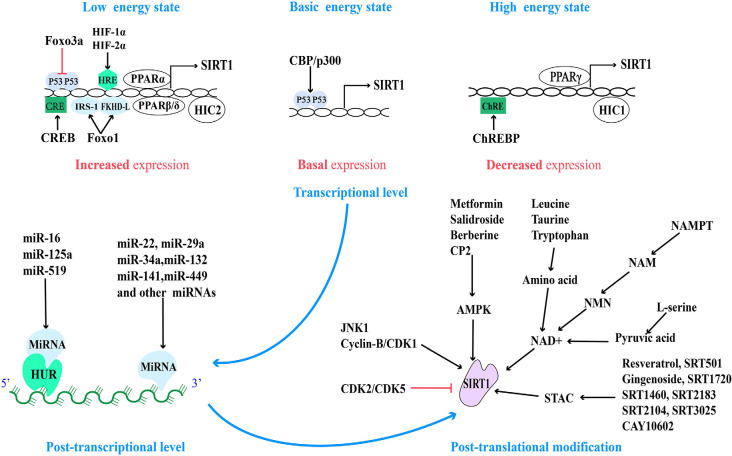
Regulatory sites for SIRT1 protein expression and activity and related pharmacological agents. Based on the different energy states of cells, the activity of the SIRT1 promoter is regulated by a variety of transcriptional regulatory factors. And at the post-transcriptional level, SIRT1 mRNA is regulated by a variety of miRNA. Finally, the activity of post-translational SIRT1 proteins is regulated by different post-translational modification mechanisms.

Conversely, under a high energy state, carbohydrate response element-binding protein (ChREBP) decreases the expression of SIRT1 by interacting with ChRE binding site on the SIRT1 promoter (Noriega et al., 2011). Another transcription factor is PPARγ, which not only can be initiated by the SIRT1-PGC-1α axis, but also PPARγ itself can directly bind to the SIRT1 promoter to inhibit the expression of SIRT1, thus PPARγ and SIRT1 compose a negative feedback loop (Han et al., 2010). Consistent with this, the hypermethylated in Cancer 1 (HIC1) negatively regulate SIRT1 expression by relying on carboxyl-terminal-binding protein (CtBP), and HIC1 also can be deacetylated and inactivated by SIRT1, thus reducing its inhibitory effect on SIRT1 expression (Paget et al., 2021). Moreover, recent studies have shown that HIC2, which is highly homologous to HIC1, is a transcriptional activator of SIRT1 due to the opposite activity of their intermediate domains, and it has been found that ectopic overexpression of HIC2 may protect the heart from ischemia-reperfusion injury (Song J.-Y. et al., 2019). In summary, it is suggested that the expression of SIRT1 is regulated by multiple negative feedback loops (Figure 2).

### Regulation by Post-transcriptional Level

MicroRNAs (miRNAs), a small non-coding RNA, can regulate the transcription or post-transcriptional regulation of protein-coding genes by degrading mRNA or inhibiting translation (Włodarski et al., 2020). The micro-regulation of SIRT1 mRNA by different miRNA has a variety of functions in different cells and tissues, including from physiology and metabolism to disease pathologies such as cardiovascular disease and cancer. These miRNAs can inhibit the transcription and expression of SIRT1 by directly binding to the 3′ non-coding region of SIRT1 or indirectly by regulating the level of human antigen R (HUR). HUR is an RNA binding protein that is usually combined with miR-16, miR-125a, and miR-519 to indirectly regulate the stability of SIRT1 mRNA (Zhang et al., 2021). Most miRNAs inhibit the expression of SIRT1 (Yamakuchi, 2012).

Nowadays, the micro-regulation of mRNA by miRNA remains a research hotspot. In addition to the high expression of miR-22, miR-29a, miR-34a, miR-132, miR-138-5p, miR-143/145, miR-195, miR-199a, miR-217, and so on in the cardiovascular system (Mao et al., 2019; Ding et al., 2020; Zhou et al., 2020), the high expression of miR-9, miR-93, miR-100, miR-122, miR-132, miR-135a, miR-137, miR-155, miR-181a/b/c, miR-182-5p, miR-199b, miR-204, miR-221, and so on in other tissues can also directly inhibit the expression of SIRT1 (Ge et al., 2019; Guo et al., 2019; Long et al., 2019; Xie et al., 2019; Zhao et al., 2019). On the contrary, miR-16, miR-125a, and miR-519 can indirectly regulate SIRT1 by regulating HUR (Yuan et al., 2016). MiR-34a was the first miRNA, to be found to regulate Sirt1, so it is the most studied. Aging and pathological conditions can increase the expression of intracellular miR-34a, thus inhibit the expression of SIRT1, and further promote aging and disease progression (Chen et al., 2019; Song L. et al., 2019; Wang L. et al., 2020). Recent studies have shown that miR-141 mimics can reduce the expression of SIRT1 mRNA, inhibit hepatocyte autophagy, reduce HBV replication (Yang et al., 2017), and promote the progress of intervertebral disc degeneration (IDD; Ji et al., 2018). Overexpression of miR-449 inhibits the osteogenic differentiation of bone marrow mesenchymal stem cells in hyperglycemia and free fatty acid microenvironment in which SIRT1 was involved, which may play a role in the fight against diabetic osteoporosis (Qu et al., 2018). Further exploration of miRNA-based therapeutic ideas can provide new intervention approaches for clinical multiple diseases (Figure 2).

### Regulation by Post-translational Modification

In addition to the above-mentioned regulation of SIRT1 activity and expression based on NAD+ level and AMPK activation, all sirtuins contain N-terminal and C-terminal domains (NTDs and CTDs), which are evolutionarily highly conserved catalytic domains, and both contain residues modified by post-translational mechanisms, of which the catalytic domain of SIRT1 is the largest (Dong, 2012). Fifteen phosphorylated residues have been identified in SIRT1, eight in CTDs, and seven in NTDs (Sasaki et al., 2008). Their phosphorylation status can be regulated by different phosphokinases. For example, under starvation (low-energy state), the enhanced cAMP signal can not only induce the phosphorylation of the highly conserved Ser434 site in the catalytic domain of SIRT1 (Hu et al., 2020), but also can be modified by intermediate phosphokinase at Ser 47,605 and 615 sites of SIRT1 and promote the dissociation of SIRT1 and DBC1, which is dependent on AMPK, then promote fatty acid oxidation, adapt to stress and meet the needs of cell energy metabolism through activating SIRT1 in an NAD+-independent manner (Gerhart-Hines et al., 2011; Nin et al., 2012).

When cell mitosis increases, cell cycle-dependent kinase (Cyclin-B/CDK1) can mediate the phosphorylation of SIRT1 at Thr530 and Ser540 to up-regulate SIRT1 activity, which is beneficial to provide energy for mitosis and promote mitochondrial division (Sasaki et al., 2008). Furthermore, c-Jun N-terminal kinase-1 (JNK1) can phosphorylate the Ser27, Ser47, and Thr530 sites of human SIRT1 and this modification of SIRT1 increased its enzymatic activity and nuclear localization (Nasrin et al., 2009). Under high glucose conditions (high nutritional availability), JNK1 phosphorylates the Ser27 site and temporarily activates the SIRT1 activity, then it is degraded through the ubiquitin-proteasome pathway, decreases the expression of SIRT1 in general (Gao et al., 2011). However, inhibition of CDK5 phosphorylation at Ser47 to modify SIRT1 not only blocks the degradation of SIRT1 through the ubiquitin-proteasome pathway (Zhang Q. et al., 2018), but also the podocyte injury and mitochondrial dysfunction in diabetic patients were significantly reduced (Wang S. et al., 2020). In addition, CK2-mediated phosphorylation of SIRT1 at the Ser164 site inhibits its nuclear localization and promotes the progress of non-alcoholic fatty liver (Choi et al., 2017), which provides evidence that SIRT1 is involved in the improvement of metabolic diseases. However, A recent study showed that in senescent cells, down-regulation of CK2 can mediate the inactivation of SIRT1, thus activating NF-KB and inducing the expression of senescence-associated secretory phenotype (SASP) factors (Song and Bae, 2021). This suggests that SIRT1 accepts negative regulation from CK2, but more evidence is needed to guide us to accurately understand the role of CK2 in different pathological models.

The strict multi-level regulation mechanism of SIRT1 is beneficial for cells to adapt to various stress states including energy stress. Post-transcriptional modification mostly regulates the activity of proteins. In light of this, the rapid regulation of SIRT1 activity may be the starting point for cells to rely on SIRT1 to adapt to stimulation, and effective regulation of this metabolic node will be of great benefit to rapid cell response. On the other hand, transcriptional activity regulation and post-transcriptional regulation play an vital role in maintaining the abundance and stability of SIRT1 protein. Due to the extensive involvement of SIRT1 in cell life activities, an in-depth understanding of these regulatory mechanisms may soon provide effective interventions for the improvement of human health (Figure 2).

## Promoting The Expression and Activity of SIRT1 Exert Beneficial Roles

At the present moment, the activation of the SIRT1 pathway has become an attractive therapeutic intervention target, and this momentum prompted researchers to actively seek SIRT1-activating compounds (STACs). The chemical nature of most STAC is polyphenolic compounds with the coplanar arrangement of hydroxyl groups on the benzene ring. In addition, some pharmacological agents can enhance the activity of SIRT1 in other ways and bring beneficial effects for diseases.

### Resveratrol

Resveratrol, a natural polyphenol compound found in the last century, as a classical activator of the SIRT1 pathway, the activation of downstream signal molecular mechanism still has not been elucidated in detail. Early studies have found that resveratrol mimics the life-prolonging effect of CR in yeast and multicellular organisms through SIRT1/Sir2-dependent pathway (Wood et al., 2004). Most of the currently recognized mechanisms are: (1) resveratrol directly interacts with the NTD domain of SIRT1, promoting the closer binding of SIRT1 to the effector protein and stimulating the activity of SIRT1 protein (Cao et al., 2015); and (2) resveratrol can also increase the level of intracellular NAD+ by activating AMPK and through NAMPT-dependent salvage synthesis pathway, and then enhance the activity of SIRT1 (Lan et al., 2017), which plays a beneficial role in energy metabolism and age-related diseases.

Injection of resveratrol into mice can not only simulate the effects of CR, such as anti-aging, promoting browning of white adipocytes, and improving metabolic disorders in mice (Li Z. et al., 2020; Pyo et al., 2020), but also play a protective role in neurodegenerative diseases such as AD, amyotrophic lateral sclerosis, and PD (Zhang L.-F. et al., 2018; Ma et al., 2019). A study has demonstrated that long-term resveratrol supplementation can improve the homeostasis between mitochondrial biogenesis and mitochondrial autophagy by activating miR-34a/SIRT1-mediated signaling pathways, and strikingly reduce age-related cochlear hair cells loss and hearing loss in mice (Xiong et al., 2019). Furthermore, accumulating scientific evidence indicates that Amyloid-β (Aβ) protein can inhibit the AMPK-SIRT1-PGC-1α pathway, inhibit mitochondrial biogenesis, and reduce glucose metabolism (Dong et al., 2016; Carbonell et al., 2020). However, it has been recently reported that resveratrol not only reduces the expression of Aβ and p-tau protein in AD model mice, increase the activity of toxic protein degradation pathway, and improve the protein balance between normal mice and AD model mice, but also up-regulate SIRT1-mediated mitochondrial biogenetic pathway and promote the recovery of mitochondrial function and energy supply by increasing the level of AMPK protein (Corpas et al., 2019). Because the new strategy to improve obese patients is to reduce the fat burden of patients by “browning” white adipose tissue to burn excess fat. And it has been demonstrated that resveratrol can activate the SIRT1-mediated white fat browning pathway and improve hyperglycemia and hyperlipidemia in mice (Li Z. et al., 2020). This may be due to the deacetylation of Lys268 and Lys293 sites on PPARγ, which can lead to the recruitment of BAT program coactivator Prdm16 to PPARγ, resulting in browning of white adipose tissue and inhibition of genes related to insulin resistance. Therefore, resveratrol has become a promising therapeutic strategy in the fight against metabolic diseases such as obesity and diabetes.

However, resveratrol did not have a lasting beneficial effect in the experiment. Researchers found that the dose of resveratrol as a key factor makes it possible to activate AMPK, in a SIRT1-dependent/SIRT1-independent manner. Moderate doses of resveratrol (25 μM) can stimulate AMPK *in vitro* and *in vivo* through the SIRT1-dependent pathway, resulting in more sustained improvement of mitochondrial function. On the contrary, a high dose of resveratrol (50μM) not only leads to the activation of AMPK in a SIRT1-independent manner, but also leads to a significant reduction in mitochondrial function and intracellular ATP level (Price et al., 2012). If the dose of resveratrol has a significant effect on the results, then the low bioavailability of resveratrol itself in humans is also one of the problems that researchers urgently need to solve. Given the rapid metabolism of resveratrol in the liver and the influence of intestinal microflora in the intestine, researchers have developed a series of drug delivery systems such as embedding resveratrol in lipid nanoparticles or liposomes, using emulsions or micelles. Resveratrol derivatives such as SRT501 have also been developed, and preparations such as resVida and Longivinex that can greatly improve the bioavailability of resveratrol have been designed (Chimento et al., 2019; Singh et al., 2019).

### Other STACs

Currently, the direction of searching for STAC is mainly extracted from natural products or artificially designed compounds according to the structure of SIRT1. In the latest screening, scientists found 19 kinds of STAC with anti-mitochondrial oxidative damage from traditional medicinal plants. Further studies on several of these activators have found that these compounds (including ginsenoside Rb2, ginsenoside F1, ginsenoside Rc, and schisandrin A) can enhance SIRT1 deacetylation activity, promote mitochondrial functional recovery by restoring oxygen consumption and increasing mitochondrial biogenesis, increase ATP production, decrease intracellular ROS levels, and strengthen antioxidant mechanisms (Wang et al., 2016).

Ginsenoside is the main active component of the pharmacological activity of Panax ginseng. Ginsenosides are classified according to different structural types, recent studies found that ginsenoside Rg3 can enhance the endurance of aged rats during exercise by affecting the activity of SIRT1 and the expression of PGC-1α (Yang et al., 2018), and six ginsenosides, including ginsenosides Rg1, Rb1, Rh2, Rg3, Rg5, and Re can protect the brain from ischemic injury by relying on SIRT1-activated signal pathways, among which ginsenosides Rb1 and Rg3 have the strongest therapeutic activity (Cheng et al., 2019). Consistent with this, as a STAC, ginsenoside Rc can promote energy metabolism in cardiomyocytes and neurons by activating the SIRT1-PGC-1α pathway to induce mitochondrial biosynthesis (Huang et al., 2021).

SRT1720 is a small molecular activator that is structurally independent of resveratrol, but its activation effect is hundreds of times that of resveratrol. It has the same effect as SRT501 and resveratrol and effectively solves the weakness of the low bioavailability of resveratrol. Resveratrol and SRT1720 bind at the same molecular site to activate SIRT1 activity and play a similar role in regulating the stability between mitochondrial biogenesis and mitochondrial autophagy. Activation of SIRT1 by SRT1720 increased mitochondrial biosynthesis *via* PGC-1α-dependent pathways, promoted the recovery of mitochondrial protein and function, and induced the protective effect of brain injury after intracerebral hemorrhage in rats (Zhou et al., 2017). SRT1720 can produce neuroprotective effects similar to resveratrol, reduce the content of Aβ in the brain of PD patients, and repair the autophagy dysfunction (Bai et al., 2021). Studies have also shown that SRT1720 plays an active role in the treatment of diabetes and insulin resistance, SRT1720 can promote wound healing and angiogenesis in diabetic mice *in vivo* and *in vitro* by activating SIRT1 (Li et al., 2019). However, studies have shown that the metabolic role of SRT1720 depends on AMPK, which is because SRT1720 cannot promote mitochondrial function and improve glucose tolerance in AMPKα 2 knockout mice (Park et al., 2017).

At present, several other STACs: such as SRT1460, SRT2183, RT2104, SRT3025, and CAY10602, have been developed (Figure 2), although there are few related studies, preliminary experiments have shown that they activate SIRT1 to mimic the anti-aging effects of resveratrol and improve the metabolic function of diabetic (type 2 diabetes) and obese mice (Zhang et al., 2018; Cheang et al., 2019). Based on this effect, new drug strategies can be provided for the treatment of metabolic disorders and age-related diseases.

However, some scholars have put forward their views on the mechanism of STAC. They reported that SRT1720 and SRT2183 can effectively reduce acetylated p53 in cells treated with DNA damage agents by inhibiting the activity of P300 histone acetyltransferase even in cells lacking SIRT1 (Huber et al., 2010), indicating that the level of acetylation *in vivo* may be affected by various factors when using STAC. Recently, scholars have revealed the epigenetic alterations in patients with AD through an integrated multi-omics approach, in which the up-regulation of histone acetyltransferase expression in H3K27ac and H3K9ac leads to an increase in their acetylation level, and the activity and expression of SIRT1 in AD patients are also decreased (Nativio et al., 2020), which leads us to wonder whether restoring histone acetylation levels in patients with AD might reverse epigenetic alterations and provide new therapeutic targets for improving AD?

### AMPK Activator

Part of the mechanism of metformin in the anti-aging and treatment of type 2 diabetes and neurodegenerative diseases is achieved by stimulating AMPK. The researchers found that AMPK activated by metformin preconditioning can effectively reduce the disorder of glucose and lipid metabolism, cellular oxidative stress, and renal function damage in diabetic rats through the SIRT1-dependent pathway, which has a protective effect on the pathological process of diabetes and diabetic nephropathy (Ren et al., 2020). In addition, the effects of metformin vary by cell and age. Metformin can activate AMPK and its downstream signal pathways in neurocytes, promote the recovery of mitochondrial function and reduce the accumulation of toxic proteins. Metformin combination therapy can even restore broken mitochondria to an almost normal state, thus meeting the energy needs of cells and improving age-related neurodegenerative diseases (Sharma et al., 2021). However, in cancer cells, AMPK activated by metformin can further activate the SIRT1/NF- κB pathway, induce mitochondrial dysfunction, and trigger cancer cell pyroptosis (Zheng et al., 2020). Furthermore, a recent study pointed out that the body’s response to metformin is affected by age. Contrary to the anti-aging effect observed in young nematodes, metformin can aggravate aging-related mitochondrial dysfunction and lead to a shortening of life span in old *Caenorhabditis elegans* (Espada et al., 2020). The observed “cell and age discrimination” of metformin once again urges us to critically review the conventional wisdom and further explore the role of metformin in aging and aging-related diseases. Metformin plays a beneficial role by activating SIRT1-mediated, AMPK-independent autophagy pathways, and also combats the inhibition of intracytoplasmic P53 on parkin-mediated mitophagy, thus maintain mitochondrial quality (Song et al., 2016). This result is similar to another study, metformin promotes the expression of mitophagy-related genes to restore parkin-mediated mitophagy pathway activity, maintain mitochondrial integrity and cell survival, protect cells from high glucose-induced damage (Zhao and Sun, 2020). However, the association between mitophagy and SIRT1 has not been fully elucidated.

Other AMPK activators such as berberine and salidroside can significantly up-regulate the expression of phosphorylated AMPK, promote mitochondrial quality, improve insulin resistance and glucose uptake through stimulating AMPK/SIRT1 and its downstream signaling pathway (Shan et al., 2020; You et al., 2020). Moreover, the tricyclopyranone compound CP2 (Zhang et al., 2015), designed to have anti-central neurotoxic proteins and other neuroprotective effects, also activates AMPK, promoting mitochondrial function and reducing toxic protein accumulation (Figure 2).

## Conclusion and Discussion

The imbalance of energy supply and demand make the body in a state of energy stress. Cells often redistribute the energy generated to important mechanisms and activities when the energy supply cannot meet the energy demand. As the main energy producer, the energy production of mitochondria is closely regulated by a variety of energy receptors in cells. Promoting the biosynthesis and function of mitochondria to produce enough ATP to meet the energy gap of cells has become an important means to break the energy stress state of cells.

SIRT1, the “hub” of intracellular signal, is widely involved in the processes of cell survival, apoptosis, inflammation, stress adaptation, cell growth and differentiation, metabolism, and senescence by regulating intracellular acetylation. However, the expression and activity of SIRT1 decreased significantly in pathological conditions such as aging or age-related diseases. In this review, we discussed that precise regulation of SIRT1 activity at all levels can eliminate cellular energy stress. We also discussed that SIRT1 molecular activator can promote the activity and expression of SIRT1 and play a useful role, which can be used as promising methods for the treatment of aging, neurodegenerative diseases, metabolic diseases, and other diseases.

As artificial energy stress can be tolerated by the body, previous studies have shown that different degrees of CR (20–50%) can cause beneficial metabolic adaptation in humans or mice, including the promotion of mitochondrial capacity and mitochondrial efficiency. Other studies also have found that a long-term CR diet can improve metabolism and promote fat loss, but the effect of fat loss will be quickly reversed once stopped (Melo et al., 2019). Therefore, I think we can implement CR with a continuous and progressive degree on mice to observe the effect on the body compared with the effect of CR with a constant degree of persistence. At the same time, the effect on weight reversal was observed by progressively reducing the extent of the CR diet rather than abruptly stopping the CR diet at the end.

NAD+ is the core prosthetic group for SIRT1 to exert its deacetylation activity, many pathways to increase SIRT1 activity are achieved by increasing the NAD+/NADH ratio. At present, some NMN-related preparations have been marketed as anti-aging products, but the effects of NAD+ metabolism have been objectively analyzed. Studies have revealed that a high NAD+/NADH ratio can drive the inflammation-related secretion phenotype of cancer-induced aging (Nacarelli et al., 2019). This is a wake-up call for the future clinical development of NAD+-enhanced supplements: we should use dietary NAD+-enhanced supplements precisely to balance their pros and cons.

On the other hand, our study found that mitochondrial function is impaired under pathological conditions, which may be partly due to changes in mitochondrial dynamics and increased mitochondrial fragmentation (Yang D. et al., 2021). The SIRT1 pathway is beneficial to restore the quality and efficiency of mitochondria, so promoting the dynamic recovery of mitochondria is expected to be achieved by promoting the biosynthesis of mitochondria. Previous studies mainly focused on the nuclear-cytoplasmic shuttle of SIRT1. The subcellular localization of SIRT1 shows that SIRT1 in mitochondria can also communicate with SIRT1 in the cytoplasm, which provides a basis for us to accurately regulate the function of mitochondria through SIRT1.

In conclusion, although the mechanisms of SIRT1 and SIRT1-related activators in anti-aging and age-related diseases have not been clarified in detail, there is a complex and inseparable relationship between the fine regulation mechanism of SIRT1 activity and expression and the mitochondria function. Therefore, it is extremely important to further clarify the causal relationship between them to provide direction for the promotion of human health and anti-aging.

## Author Contributions

YF, JY and FH: conception, designing, and writing of the manuscript. XW, JY, DY, WY, XL and QZ: assisted in literature search, conception, and writing of the manuscript. FH, YL and GW: conception and revising the manuscript. All authors contributed to the article and approved the submitted version.

## Conflict of Interest

The authors declare that the research was conducted in the absence of any commercial or financial relationships that could be construed as a potential conflict of interest.

## Publisher’s Note

All claims expressed in this article are solely those of the authors and do not necessarily represent those of their affiliated organizations, or those of the publisher, the editors and the reviewers. Any product that may be evaluated in this article, or claim that may be made by its manufacturer, is not guaranteed or endorsed by the publisher.
